# Application of 3D printed pelvic fracture related urethra and surrounding tissue as preoperative planning model

**DOI:** 10.1186/s12894-022-01165-7

**Published:** 2023-01-06

**Authors:** Kaile Zhang, Jiafu Liu, Liyang Wang, Wenyao Li, Er Qi, Qiang Fu

**Affiliations:** 1 The Department of Urology, Affiliated Sixth People’s Hospital, Shanghai Jiaotong University School of Medicine, No. 600, Yishan Road, Shanghai, China; 2 School of Materials Science and Engineering, Shanghai University of Engineering Science, Shanghai, China; 3 Shanghai Xietu Community Healthcare Center, No. 600, Yishan Road, Shanghai, China

**Keywords:** Urethral stenosis, 3D printing, Pelvic fracture urethral injury, Urethral plasty

## Abstract

**Objective:**

Urethral stenosis caused by pelvic fracture urethral injury (PFUI) is a complex urological disease, especially for the redo cased. However, to find the proximal end of the posterior urethra, and to avoid injury to the rectum and to forecast to remove the inferior pubic margin are two key points for a successful surgery. These steps can be challenging for even the most experienced urologists. This study is to describe a new technique for understanding the three-dimensional (3D) anatomy of the urethra, which will also aid in surgical planning and simplify urethroplasty.

**Materials and methods:**

Three patients underwent routine urethroscopy, X ray urethrography and contrast CT urethrography. The 3D images were then reconstructed, and the data were transmitted to a 3D printer. 3D models were printed with polyacrylic acid to simulate the anatomical structure and relationship of urethral stenosis with pubic symphysis and rectum. Various diagnosis methods were compared with the condition in surgery. The patients and trainee questionnaires were performed.

**Results:**

Three models of urethral CT were obtained. These models were presented to patients and trainee doctors along with routine urethroscopy, urethrography, and urethral CT. The scores of patients and trainee question forms demonstrated that the 3D printed urethral stenosis model of pelvic fracture has obvious advantages in urethral adjacency and ease of understanding. The 3D printed urethras were easy to show the pubic symphysis and simulate its excision and exposure of urethra. The model could show the precise distance from urethra to rectum to prevent the rectum injury in surgery.

**Conclusions:**

3D printing technology can be applied to the preoperative evaluation of urethral stenosis caused by PFUI. It can be auxiliary to understand the anatomical structure of the posterior urethra, the direction of urethral displacement, protecting the rectum and the forecasting for pubectomy. It is especially helpful for the accurate preoperative planning of some complex urethral stenosis and redo cases.

## Introduction

Pelvic fracture urethral injury (PFUI) is the most common type of traumatic urethral stenosis. Due to the particularity of the local tissue anatomy of the posterior urethral region, such as the lack of cavernous tissue supporting free substitutes, the poor blood supply of the pedicled skin flap, denser scar tissue, and the deep position of the healthy urethra, which make it difficult to treat this kind of stricture with urethral substitutes [1, 2]. Although posterior urethral anastomosis has been relatively mature, the management of complex long posterior urethral stenosis, such as urethral gap (> 4 cm), especially with multiple surgical failures, bladder floatation, urethral stenosis and urethrorectal fistula, has always been the most difficult problem in urethral reconstruction [3].

Traditional preoperative evaluation includes urethroscopy and urethrography. For some complex cases, CT urethrography and magnetic resonance urethrography may be used. Urethral ultrasound has high requirements for operators and is not widely popularized [4–6]. There is no general image of urethral stenosis in urethroscopy. At the same time, the imaging mode of urethrography is a two-dimensional image under X-ray transmission. It is not only unable to accurately judge the length of urethral stenosis, but also difficult to correctly predict the auxiliary operation required in posterior urethral anastomosis, nor can it show the position relationship between urethra and rectum [7]. In pelvic fractures, the urethra may be pulled backward to the rectum, resulting in rectal injury during operation, or pulled forward to the pubic bone. The urethra was blocked by the pubic symphysis and cannot be connected. The lower edge of the pubic bone needs to be removed during the operation, and sometimes the two situations occur at the same time. In complex cases, CT or MRI can be used to evaluate the proximal urethra, but it is still not intuitive enough, and there are high requirements for film reading experience, which makes it difficult to assist the operation, let alone effectively carry out patient education [8].

In this study, we describe a new three-dimensional (3D) printing method to determine this complex anatomical 3D view, which is also helpful to predict the subsequent surgery steps and auxiliary operation methods.

## Materials and methods

We conducted a pilot study involving 3 patients with PFUI. This study only used the CT urethrography image data of the patients, and obtained the written informed consent of the patients. No patient incurs any cost due to CT scanning or 3D printing.

Three patients with failed urethral anastomosis after 6 months were analyzed. The all underwent CT scanning to evaluate the anatomical relationship between the two ends of the urethra. The bladder was filled with sterile normal saline and radioactive contrast agent through the suprapubic vesicostomy channel (50 ml meglumine diatrizoate injection was diluted into 200 ml sterile normal saline). Fill the anterior urethra with the same contrast medium solution, and ask the patient to hold the penis tightly to retain the contrast medium. Plain CT scan of pelvis was performed from the swollen bladder to the penis. Then CT images are processed in standard 3D reconstruction software. Then 3D printer was used to reconstruct 3D printed anatomical model. It uses polycaprolactone fiber material to reconstruct the 3D printing model, which takes an average of 5 h. This method was used in all 3 patients in this study.

This method is used for patient and doctor’s education. The patients score by themselves. Comparing its comprehensibility with urethroscopic, urethrography, and urethral CT The patients score 1 point for difficult understanding, 2 points for understanding and 3 points for easy understanding. The doctor’s score includes judging whether there is stenosis, displaying the length of stenosis, the relationship between urethra and pubic symphysis, and the relationship between urethra and rectum. If the display is poor, it will be 0 point, if it can be displayed, it will be 1 point, if it is better, it will be 2 points, and if it is good, it will be 3 points. Summarize the scores for statistics.

Various diagnosis methods were compared, including urethrogram, urethroscopy, urethral CT, 3D printing, and the real conditions in surgery. The defect lengths were measured from the proximal to the distal urethra. The distances from the proximal urethra to the rectum were measured. The pubic symphysis margins of 3D printed models were excised to expose the proximal urethra. The length and width was measured.

## Results

**Fig. 1:**
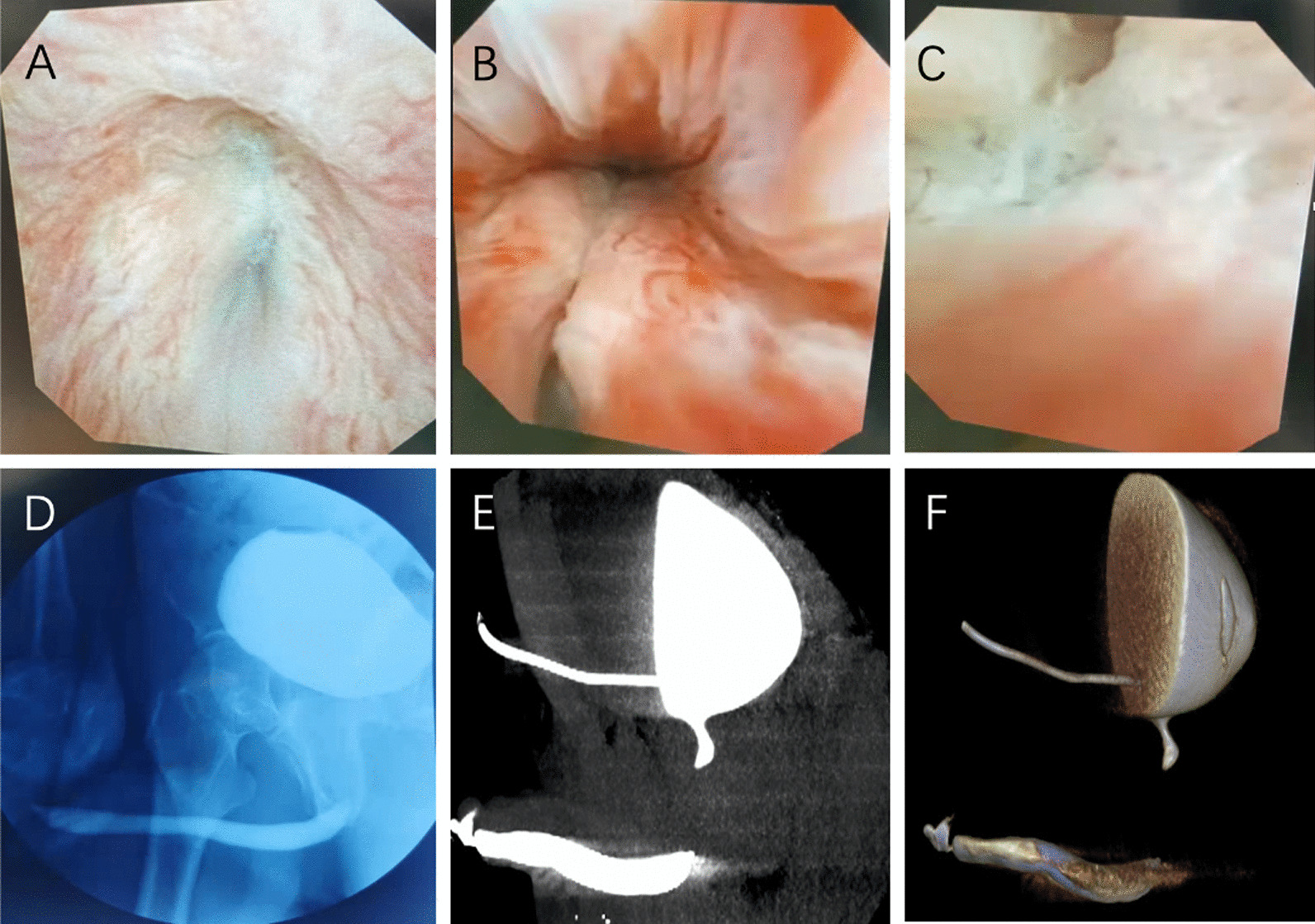
Various methods of urethral evaluation **A** distal end of urethra in patients with urethral stenosis under urethroscope; **B** Bladder neck of patients with urethral stenosis under urethroscope; **C** Proximal end of urethra in patients with urethral stenosis under urethroscope; **D** Urethrography showed urethra and bladder; **E** CT scan of urethra and bladder in sagittal plane; **F** 3D reconstruction of the urethra and bladder from CT scans

**Fig. 2:**
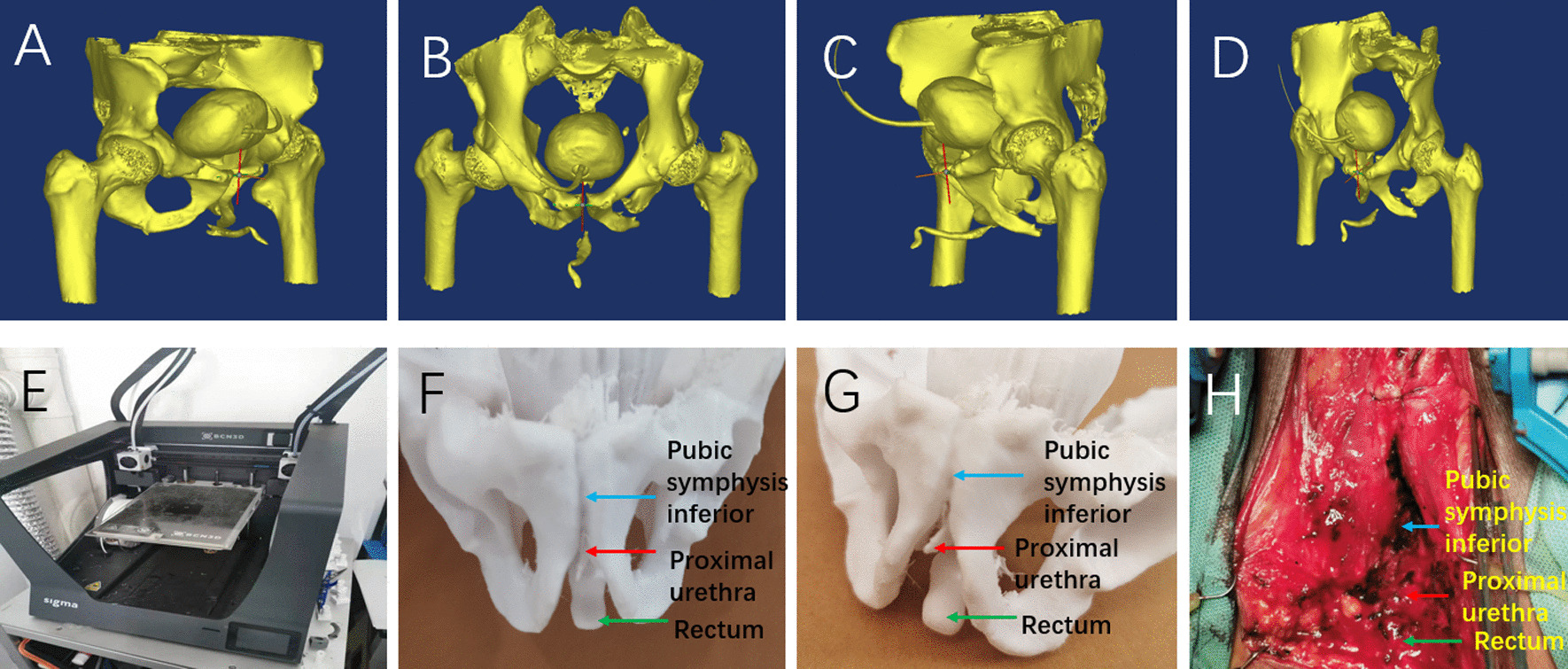
**A** right lateral view of pelvis and bladder urethra reconstructed by computer; **B** front view of pelvis, bladder and urethra reconstructed by computer; **C** left side view of pelvis and bladder urethra reconstructed by computer; **D** top view of pelvis, bladder and urethra reconstructed by computer; **E** 3D printer for pelvic and urethral model construction; **F** Front view of 3D model of pelvis and urethra; **G** Left lateral view of three-dimensional model of pelvis and urethra; **H** the structure of urethra and its adjacent relationship during operation suggest that pubectomy is necessary

Three patients with complete urethroscopy, urethrography and urethral CT were enrolled in this study. The image data were used as the control group (Fig. 1). Through 3D reconstruction of computer image and 3D printing of pelvis and urethra model, we can understand the anatomical structure and adjacent relationship of posterior urethra. These images describe the exact location of the posterior urethra, including its relationship to the pubic bone, to the rectum, and the displacement of the midline (Fig. 2). Under the guidance of 3D printed urethral model, we conducted preoperative education and operation guidance for patients.

We showed the patients’ preoperative urethroscopy, urethrography and 3D printing model to 5 trainee doctors who came to our hospital for study. Ask whether these models help them to assess the need for inferior pubectomy for tension-free urethral anastomosis before operation. The opinions of 5 advanced doctors showed that the method of 3D printing model was feasible, and the anatomy studied on the 3D model before operation was helpful to shorten the operation time.

In terms of patient understanding score, the three patients had a better understanding of the condition in the preoperative conversation, the anatomical position of the urethra, the complexity of the operation, and the score was the highest among several methods (9 points). Five trainee doctors observed the 3D printed pelvis and urethra model, compared it with urethroscopy, urethrography and urethral CT, and compared it with the real anatomical structure during operation. They believed that the advantage of 3D printed pelvis and urethra model was that it could more accurately reflect the relationship between proximal urethra and pubic symphysis (15 points), and the relationship between urethra and rectum (12 points). At the same time, it also points out some shortcomings compared with the traditional methods, such as judging whether there is stenosis (15 points), and the length of stenosis (10 points) is inferior to that of urethroscopy and urethrography (Table 1).

**Table 1:** The urethral model scoring table for doctors and patients

	Patient understandability(n = 3)	Judge the stricture (n = 5)	Accurate evaluation of stricture length (n = 5)	Pubic symphysis relationship (n = 5)	Rectal relationship (n = 5)
Urethroscope	7 (2,2,3)	15 (3,3,3,3,3)	5 (1,1,1,1,1)	0	0
Urethrography	6 (2,2,2)	12 (3,2,3,2,2)	10 (2,2,2,2,2)	5 (1,1,1,1,1)	0
CT of urethra	3 (1,1,1)	9 (1,2,2,2,2)	9 (2,1,3,1,2)	10 (2,2,2,2,2)	8 (1,2,3,1,1)
3D printing	9 (3,3,3)	5 (1,1,1,1,1)	5 (1,1,1,1,1)	15 (3,3,3,3,3)	12 (2,3,2,3,2)

The age of three male patients were 42, 46 and 54 years old (Table 2). All of them had undertaken previous urethral anastomosisbut without pubic symphysis exisicion. The 3D printed model showed a disconnected urethra, so the defect length is not applicable to measure. The distance from proximal urethra to rectum is 0.5 cm in 2 patients and 0.6 cm in 1 patient in 3D printed model, it’s consistent with the CT scanning data. The images in urethroscopy, urethrogram were not feasible to evaluate the distance between urethra and pubic symphysis/rectum. In the surgeries, it was not easy to judge the distance from urethra to rectum, in spite that the digital examinations could palpate the rectum. The simulated excision of superior pubic symphysis was conducted in 3D models. Two patients had undertaken the pubic symphysis excision in surgeries to expose the proximal urethras, which were consistent with the forecasting of 3D printed models. The amount of bone fragments was similar between that in surgery and 3D printed models.

**Table 2:** The patients data and surgery information

Variables	N
Age (years)	46 (42–54)
Previous surgery before anastomosis	
Yes	3
Pubic symphysis exision	
No	3
Etiology	
PFUI	3
Defect length	(cm)
Urethrogram	2.2 (1.8–2.5)
Urethroscopy	N/A
CT	2.4 (1.9–2.7)
3D printing	Disconnected
Surgery	2.5 (2–3)
Distance from proximal urethra to rectum	(cm)
Urethrogram	N/A
Urethroscopy	N/A
CT	0.5, 0.6, 0.5
3D printing	0.5, 0.6, 0.5
Surgery	N/A
Pubic symphysis incision volume	Length, width, depth (cm)
Urethrogram	N/A
Urethroscopy	N/A
CT	N/A
3D printing	1*1*1,0,0.8*0.8*0.8
Surgery	1*1*1,0,1*1*1

*N/A* not applicable

## Discussion

In this study, we introduced a new method for the diagnosis and evaluation of urethral stenosis caused by pelvic fracture and urethral injury. Compared with urethroscopy, urethrography and urethra CT commonly used in clinic, 3D printing has its unique advantages [9, 10]. 3D printing has been tried in precision instrument parts, artificial limbs and personalized implants. A wide range of materials can be used for 3D printing, including metals, hydrogels, etc. However, the most stable 3D printing material available at present is polycaprolactone [11, 12].

In medical applications, most 3D printers use the DICOM format file after CT scanning to reconstruct the printing model [13]. Medical 3D printing model can imitate the anatomical structure in reality in its original size and can also be reduced to a miniature model. Reducing the size of the model can reduce the cost and time of printing. In this study, we used 1:2 size reduction to save printing time and facilitate rapid clinical use.

PFUI repair is one of the most complicated operations performed by urologists in the world. Transperineal urethrostomy is the best operation for urethral stenosis of pelvic fracture. It mainly includes three main steps: resection of scar tissue, exposure and repair of distal and proximal anastomosis, and tension-free urethral anastomosis. It is the standard method for the treatment of posterior urethral stenosis. However, in patients with complex long segment posterior urethral stenosis, large defect segment often forms between anastomotic stomas [14]. It is difficult to achieve tension-free anastomosis by simply pulling the distal and proximal ends, which is one of the important reasons for the failure of urethral anastomosis. It is often necessary to use the methods such as resection of the lower edge of the pubis. One of the main advantages of the 3D printing model is that it helps to accurately evaluate these cases before surgery for better preoperative planning, including the evaluation of operation time, operation difficulty and risk. In the future, we can consider using materials similar to bones for 3D printing of pubic symphysis. This will help urologists to perform pubectomy on 3D models before surgery and plan the resection scope, which may further reduce the operation time, improve the operation effect and reduce complications.

Another important advantage of 3D printing urethral stenosis model of pelvic fracture is that it can fully show the relationship between urethra and rectum. Rectal injury is severe complication in posterior urethral reconstruction, and it is also an important factor limiting the operation of surgeons [15]. Different from pure visual judgment in CT images, 3D printing can help doctors perceive the positional relationship between the urethra and rectum at the same time visually and tactilely, so that they can be confident before surgery, so as to know the direction of surgical approach, avoid rectum and significantly reduce the incidence of surgical complications. The mechanism of MRI application in urethral stenosis is similar with the CT. But the cost of MRI is much higher than CT and the scanning time of MRI is also long. at present, we just use MRI in some specific researches [16, 17].

The limitation of this study is the small cases number. In the future, it could set the postoperative effects of traditional surgery as the control group, and the postoperative effects of 3D printing assisted surgery as the test group to compare the operation time, postoperative complications and other indicators. In previous Joshi et al. reported method, who firstly introduced the method, had used acrylic fibers to reconstruct the 3D printed model [18]. In our study, the polycaprolactone fiber as a 3D printing material showed lower cost and more available 3D printers for hospitals. The present study focused on the subjective feeling of patients and trainee doctors. It had qualified the influence of the 3D printed model. From the vision of trainee doctors, they obtained more information from 3D models. In addition, we compared the 3D printed model with three traditional evaluation methods and gave a qualified data of their advantage and disadvantage. It might be beneficial for the following studies about this topic.

In conclusion, 3D printing of PUFI urethral stenosis using CT scanning data is feasible. It can be used as a patient education tool and training tool for urologists, furthermore, it can also be used for preoperative planning of complex cases.

## Data Availability

The data that support the findings of this study are available from Shanghai sixth people's hospital but restrictions apply to the availability of these data, which were used under license for the current study, and so are not publicly available. Data are however available from author Kaile Zhang upon reasonable request and with permission of Shanghai sixth people's hospital.

## Abbreviations

3D: Three-dimensional
CT: Computed tomography
PFUI: Pelvic fracture urethral injury
MRI: Magnetic resonance imaging
DICOM: Digital imaging and communications in medicine
